# Dairy farm-workers’ knowledge of factors responsible for culling and mortality in the Eastern Cape Province, South Africa

**DOI:** 10.1007/s11250-021-02845-6

**Published:** 2021-07-12

**Authors:** Yanga Simamkele Diniso, Ishmael Festus Jaja

**Affiliations:** 1 Department of Livestock and Pasture Sciences, University of Fort Hare, Alice, 5700 South Africa; 2 Risk and Vulnerability Science Centre, University of Fort Hare, Alice, 5700 South Africa

**Keywords:** Milk production, Mastitis, Culling, Dairy cows, Knowledge and attitudes

## Abstract

Milk serves as a significant source of protein for many families and aids in combating food insecurity. However, the demand for milk and milk-related products far exceeds the supply. The objective of the study was to evaluate dairy farm-workers’ knowledge of factors responsible for culling and mortality of dairy cows in the Eastern Cape Province. Data was collected from 106 dairy farm-workers using a questionnaire. Any correctly answered question by the majority amounted to a point and a zero for incorrectly answered questions. Correct answering by the majority to more than half the questions of a subsection amounted to a pass. A less than 50% pass rate was considered a poor level of knowledge, 51–69% pass rate was considered an average level of knowledge, and anything higher than that was considered a good level of knowledge. Most farm-workers (66.0%) relied on their colleagues for dairy health information. Most dairy farm-workers (49.1%) indicated that lameness, milk fever (56.6%), and mastitis (47.2%) do not lead to culling and mortality of dairy cows. A majority (83%) of farm-workers agreed that reproduction problems, poor milk yield (77.3%), and age (81.1%) are the main reasons for culling dairy cows. The participants had varying perceptions and limited knowledge (28.3%) about the major contributing factors of culling and mortality. The lack of training courses and minimal use of other sources of information such as the internet might contribute to this poor knowledge and perceptions.

## Introduction

Milk is an essential commodity in South Africa as it provides protein and other essential minerals (Ndambi and Hemme 2009; DAFF 2018). Milk is one of the animal-sourced foods that is a necessary core diet for proper childhood development and the elderly (Choudhury and Headey 2018). Hence, the dairy industry is an important sector of public health interest that produces milk for direct and indirect human consumption (Lucey 2015). In South Africa, 98% of milk from dairy farms is sent to the market, and the remaining 2% is for on-farm consumption (Milk SA 2017; MilkSA 2019). In addition, the dairy industry is one of the critical sectors used in combating food and nutritional insecurities, especially in the African continent (Semba et al. 2016; Choudhury and Headey 2018).

In addressing the malnutrition challenges in the Southern African region, milk and milk-related products provide the required cellular growth fatty acids (Semba et al. 2016; Agriculture Research Council 2017; Choudhury and Headey 2018). All these milk-related benefits have resulted in an escalation in demand for milk. Due to the regular increase in population, the demand for milk has escalated beyond the local milk producer’s capability to adequately meet the demand (Thornton 2010; Agriculture Research Council 2017, 2018; Lemmer 2018).

The dairy industry is plagued by several factors that limit its potential such as diseases, human factors, technological innovations, and environmental factors (Jaccard and Blanton 2005; Jansen et al. 2009; Barkema et al. 2015; Sumon et al. 2017). Diseases are described as key contributors to livestock production inefficiencies (Fitzpatrick 2013). Conditions such as mastitis and lameness result in significant milk quantity and milk quality losses (Hernandez-Mendo et al. 2007; Ndambi and Hemme 2009; Barker et al. 2010; Barkema et al. 2015; Der Leek 2015). Also, mastitis is a predisposing factor for culling and mortality of dairy cows (Gröhn et al. 1998; Guha et al. 2012; Humayun Kabir et al. 2017).

Mastitis is a condition of the udder with economic consequences for dairy production often caused by *Staphylococcus aureus*, *Shiga-toxin*-producing *Escherichia coli* (STEC), *Streptococcus* spp, *Klebsiella *spp, and *Mycoplasma species* (Bar et al. 2007; Islam et al. 2012; Marimuthu et al. 2014; Sumon et al. 2017). Farmer’s understanding of the main reasons for culling and cow mortality will aid in proper development and implementation of mitigation strategies of culling and mortality of dairy cows and mastitis prevention plans (Jaccard and Blanton 2005; Jansen et al. 2009; Pexara et al. 2018). Hence, this study seeks to evaluate dairy farm-workers’ knowledge of factors responsible for culling and mortality of dairy cows in the Eastern Cape Province (ECP), South Africa.

## Materials and methods

### Ethical considerations

Ethical clearance certificate REC-270710-028-RA Level 1 with project number JAJ011SDINO1 was obtained from the University of Fort Hare Research and Ethics Committee (UFH-UREC) before the data collection process.

### Research design and study area

The study was conducted following a cross-sectional research design (Bryman 2012). The study was conducted purposively on dairy farms located in the south-eastern part of the ECP in five districts out of the province’s six districts. The districts include Amathole, Chris Hani, OR Tambo, Cacadu, and Alfred Nzo. The ECP is the second largest province in South Africa and home to approximately 6,500,000 people. The province occupies 169,580 km^2^ (13.9%) of the country’s total land area, with about 63% of rural areas plagued with a high unemployment rate. The ECP is the second highest producer of unpasteurised milk in South Africa, contributing 30% of the country’s overall milk production (DAFF 2018; Milk Producers Organisation 2018; MilkSA 2019). Besides, the ECP has the highest number of cows in milk averaging 760 cows per farm than any other province.

### Study population

**Fig. 1:**
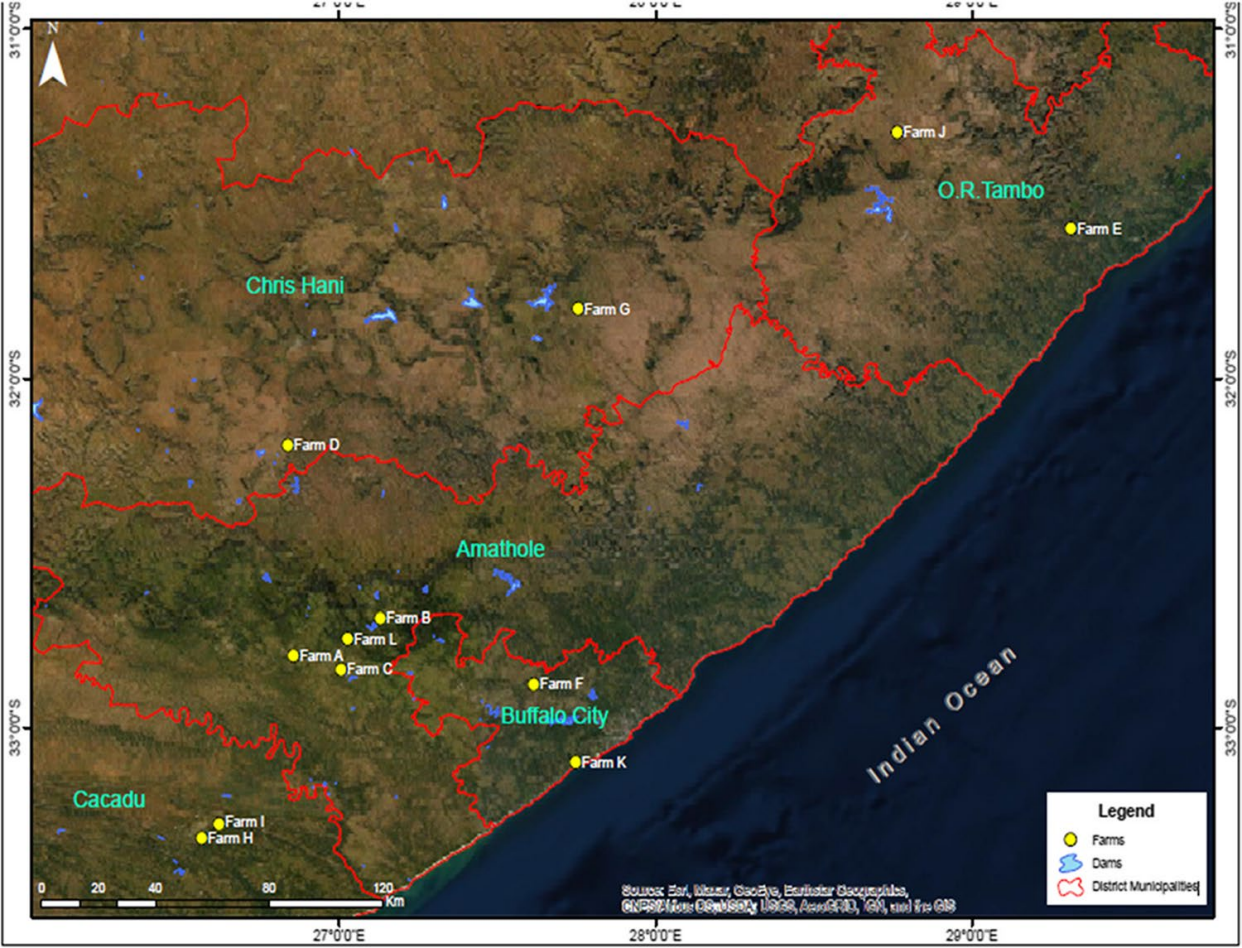
The Eastern Cape Province map depicting the districts and the study sites

Twenty dairy farms were randomly selected, telephonically approached, and sent emails requesting permission to visit and conduct the research. A snowball technique was used to reach out to small-scale dairy farms. Approval to conduct the study was obtained from 12 dairy farms, and these were the farms included in the final survey (Fig. 1). Approximately 20 dairy workers per farm were targeted, including the managers, supervisors, general workers, and bulk-tank workers. However, on average, there were only 5–10 milkers per milking session in each dairy farm, varying with farm sizes. As such, 106 respondents out of a possible 120 (10 from 12 dairy farms) correctly completed the questionnaire. Nine incorrectly filled questionnaires were excluded from the analysis.

### Data collection

A questionnaire was developed, validated, and piloted on the nearby dairy farm-workers and students undertaking practical training on the farm. Piloting was done to note the ease of answering the questionnaire and even the time it takes to fill it. The questionnaire purposively consisted of close-ended questions, thus generating quantitative data (Bryman 2012). The questionnaire was designed in English, but it was translated as per the respondent’s home language during data collection. Open-ended questions provided a platform for respondents to supply any additional information of value to the study. Demographic information, sources of information about dairy health, and knowledge and perceptions about culling and mortality data were collected from the farm-workers. There was very limited susceptibility to social-desirability biasness of the results (McConnel et al. 2017).

### Statistical analysis

The questionnaire data was coded in Microsoft Excel for quantitative analysis (Bryman 2012). Descriptive statistics analysis was performed with IBM SPSS Statistics 25 to identify frequencies for demographics and associations amongst nominal data variables. Any correctly answered question by the majority amounted to a point and a zero for incorrectly answered questions (Moreb et al. 2017). Correct answering by the majority to more than half the questions of a subsection amounted to a pass. A less than 50% pass rate was considered a poor level of knowledge, 51–69% pass rate was considered an average level of knowledge, and anything higher than that was considered a good level of knowledge. Cronbach’s alpha based on standardised items was generated to test reliability, which amounted to 0.923. Chi-square (*X*^2^) test was adopted to test for statistical associations amongst variables. In a case whereby *P* ≤ 0.05, the findings were regarded as significant.

## Results

Most of the respondents were males, 60%, while females accounted for 40% of the study population (Table 1). Most participants (49.1%) were aged between 21 and 30 years, and 13% were aged 41–60 years. About 45% of the respondents had a matric certificate, and 32% had no basic formal education. Furthermore, 45.3% of the respondents had 0–3 years of dairy experience, and 32% had more than 5 years of dairy experience. Most respondents (59%) were general workers, and 23% were farm managers. The results also revealed that 57% of the respondents had dairy herd health training, with the remaining having no training.

**Table 1:** Demographic profile

Demographic characteristics	N	Category	Frequency	Percentage
Gender	106	Female	42	39.6
		Male	64	60.4
Age	106	Below 20 years	4	3.8
		21–30	52	49.1
		31–40	36	34.0
		41–60	14	13.2
Workplace position	106	Manager	24	22.6
		Supervisor	4	3.8
		General worker	62	58.5
		Temporary worker	16	15.1
Educational level	106	Less than grade 12	34	32.1
		Grade 12	24	22.6
		Above grade 12	48	45.3
Work experience	106	0–3 years	48	45.3
		4–5 years	24	22.6
		Above 5 years	34	32.1
Marital status	106	Single	84	79.2
		Married	22	20.8
		Divorced	0	0
		Widowed	0	0
Tribe	106	Black	98	92.5
		White	6	5.7
		Coloured	2	1.9
		Indian	0	0

Majority of respondents (62.3%) reported that training was the common source of dairy health information (Table 2). Additionally, most respondents (66%) use their colleagues as their primary source of information (Table 2). However, 84.9% of farm-workers mentioned that they do not use mass media as their source of information. There was a statistical relationship (*P* ≤ 0.05) between workplace position, work experience, and use of training as a source of information (Table 3).

**Table 2:** Respondents’ sources of information about dairy health and food safety information

Questions	N	Category	
What are your sources of information for dairy health and food safety?	106	Yes (%)	No (%)
Training courses	106	66 (62.3)	40 (37.7)
Mass media	106	16 (15.1)	90 (84.9)
Internet	106	30 (28.3)	76 (71.7)
Friends	106	14 (13.2)	92 (86.8)
Colleagues	106	70 (66.0)	36 (34.0)
Family	106	10 (9.4)	96 (90.6)

**Table 3:** Association between demographic features and attending course in dairy health, sources of information, and common on-farm practices

Demography	Training course	Mass media	Internet	Friends	Colleagues	Family
Age	0.021*	0.010*	0.000*	0.210*	0.181	0.234
Gender	0.115	0.016	0.406	0.150	0.074	0.006*
Workplace position	0.001*	0.827	0.002*	0.156	0.190	0.131
Educational level	0.396	0.304	0.000*	0.589	0.279	0.853
Work experience	0.019*	0.171	0.000*	0.020*	0.980	0.001*
Marital status	0.068	0.026*	0.025*	0.522	0.201	0.951
Tribe	0.520	0.463	0.648	0.518	0.000*	0.637

^*^Significant at *p* ≤ 0.05

Abortion (47.2%) was indicated as a main reason for culling and mortality of dairy cows. A total of 34% of farm-workers reported that lameness leads to dairy cows’ culling and mortality. However, 56.6% of respondents indicated that milk fever does not lead to dairy cows’ culling and mortality. Most respondents (81.1%) answered that bloat and diarrhoea do not lead to dairy cows’ culling and mortality. According to the respondents, reproduction problems (83%), poor milk yield (77.3%), and age (81.1%) are the main reasons for culling dairy cows (Tables 4 and 5). Furthermore, Table 6 shows a relationship (*P* ≤ 0.05) between respondents’ position, experience, educational level, and perception of poor milk yield and reproduction failures as the main culling reasons.

**Table 4:** Respondents’ knowledge and perceptions about mastitis; culling and mortality

Questions	N	Yes (%)	No (%)	I don’t know (%)
A cow detected with mastitis should be treated	106	97 (91.5)	2 (1.9)	6 (5.7)
Which of the following diseases are commonly responsible for culling and mortality? Abortion?	106	**50 (47.2)**	36 (34.0)	20 (18.9)
Lameness	106	**36 (34.0)**	52 (49.1)	18 (17.0)
Mastitis	106	**50 (47.2)**	50 (47.2)	6 (5.7)
Dystocia	106	**32 (30.2)**	58 (54.7)	16 (15.1)
Milk fever	106	**28 (26.4)**	60 (56.6)	18 (17.0)
Bloat	106	14 (13.2)	**86 (81.1)**	6 (5.7)
Diarrhoea	106	10 (9.4)	**86 (81.1)**	10 (9.4)
Acidosis	106	16 (15.1)	**68 (64.2)**	22 (20.8)
*Pass rate				2.26 (28.3%)

Correct answers are highlighted in bold. *Pass rate has been counted by the addition of percentages in which most of respondents correctly answered the question and divided by 100

**Table 5:** Evaluation of perceptions about factors responsible for culling dairy cows

Questions The main reasons for culling dairy cows are	N	Strongly agree	Agree	Neutral	Disagree	Strongly disagree
Reproduction problems	106	56 (52.8)	32 (30.2)	8 (7.5)	4 (3.8)	2 (1.9)
Poor milk yield	106	40 (37.7)	42 (39.6)	10 (9.4)	6 (5.7)	8 (7.5)
Diseases	106	32 (30.2)	46 (43.4)	14 (13.2)	6 (5.7)	8 (7.5)
Age	106	42 (39.6)	44 (41.5)	10 (9.4)	8 (7.5)	2 (1.9)
Poor body condition	106	18 (17.0)	34 (32.1)	18 (17.0)	26 (24.5)	10 (9.4)
Poisoning	106	14 (13.2)	28 (26.4)	38 (35.8)	18 (17.0)	8 (7.5)
Heat stress	106	8 (7.5)	20 (18.9)	20 (18.9)	36 (34.0)	22 (20.8)

**Table 6:** Association between demographic features and knowledge of common causes of culling and mortality of dairy cows

Demography	Abortion is responsible for culling and mortality	Diseases	Poor milk yield	Age	Heat stress	Reproduction failures	Lameness	Poor body condition	Mastitis
Age	0.000*	0.000*	0.000*	0.000*	0.005*	0.099	0.000*	0.001*	0.000*
Gender	0.005*	0.034*	0.166	0.232	0.029*	0.329	0.706	0.055*	0.006*
Workplace position	0.014*	0.006*	0.001*	0.001*	0.269	0.011*	0.006*	0.662	0.084
Educational level	0.001*	0.081	0.020*	0.141	0.000*	0.153	0.047*	0.002*	0.019*
Work experience	0.055*	0.032*	0.001*	0.034*	0.009	0.024*	0.507	0.033*	0.002*
Marital status	0.000*	0.001*	0.001*	0.359	0.086	0.006*	0.016*	0.020*	0.453
Tribe	0.224	0.511	0.037*	0.724	0.159	0.700	0.449	0.003*	0.475

^*^Statistically significant at *p* ≤ 0.05

## Discussion

The high number of young farm-workers in this study is different from previous youth under-representation reports in the agriculture sector (Beccio 2014; Andeweg and Lee 2020). The study is further supported by a recent study that reported considerable youth involvement in dairy farming (Galloway et al. 2018). Evidence from the current study strongly suggests a growing interest of youth in dairy production. Several factors can be accountable for these findings (high youth representation), such as high youth unemployment, career change, and exposure to scientific information about dairy farming, and the introduction of technology into the industry. The use of digital devices to monitor dairy cows’ dynamics can quickly fascinate the youth upon exposure and attract them into the dairy industry. Furthermore, dairy farming has vast job opportunities; as such, the global high youth unemployment may be a driver for young people’s interest in dairy farming (Andeweg and Lee 2020). Hence, working on a dairy farm becomes a logical option for the youth.

The high youth representation closely relates to the higher educational level (grade 12 and above) of the dairy farm-workers, which is expected to contribute significantly to the level of knowledge of the farm-workers (Weir 1999; Oduro-ofori et al. 2014). Besides, 67.9% of the respondents had a grade 12 certificate and above, which far exceeds the previously reported 17.1% of dairy workers with similar educational levels in South Africa (Stats SA 2014). The high level of education of dairy farm-workers reported in the current study coincides with a recent survey conducted in the same province but in a different region not sampled in this study (Galloway et al. 2018). The level of education of farm-workers in this study suggests an increasing interest in young people for agriculture. A higher education level is closely related to high knowledge levels and thus increased productivity of dairy farms (Weir 1999; Gasperini 2003). Therefore, the increase in knowledge levels amongst dairy farmers in recent years can be credited for the dairy industry’s recent growth in the province (DAFF 2018; Milk Producers Organisation 2018; MilkSA 2019).

The poor level of knowledge exhibited by farm-workers in this study may be due to poor and inadequate sources of dairy information used, and selective dissemination of information on the farm. Dairy farm owners/managers have the liberty to cull dairy cows (Orpin and Esslemont 2016; Compton et al. 2017). Such decisions to cull animals are often taken without the knowledge of low-level farm-workers. Hence, these farm-workers may be unaware of the reasons for culling and mortality of dairy cows. Also, dairy farm-workers’ attitude to learning may likely influence their level of knowledge. Some farm-workers often focus only on milking the dairy cows and show no interest in understanding other dynamics. The current study findings echo the previously reported assertion that high educational level of farm-workers cannot be necessarily translated to dairy farming knowledge (Appleton and Balihuta 1996; Lockheed et al. 1999; Reimers and Klasen 2012). This is because even though most dairy farm-workers were had matric and above, they still did not know the main reasons for culling and mortality of dairy cows.

The assertion of the dairy farm-workers that reproduction failures such as infertility, abortion, and non-pregnancy are the main reasons for culling and mortality of dairy cows is supported by a recent study that reported reproduction failures as a prominent reason for culling of dairy cows (Kerslake et al. 2018). Reproduction failures accounted for 60% of the costs incurred in dairy farms and categorised under farm wastages (Kerslake et al. 2018). Previous studies cited infertility as a leading risk factor of the culling of dairy cows (Bascom and Young 1998; Ghaderi-Zefrehei et al. 2017). The dairy farm-workers’ well-informed perception can be linked to the general understanding of the association of reproductive failures to milk production losses. Also, reproductive failures are regarded as a delicate matter; as such, most dairy farms conduct training and hire veterinarians for reproduction-related issues like oestrus detection, pregnancy diagnosis, and artificial insemination activities (Kerslake et al. 2018). Dairy farms prioritise the continuous monitoring of cows’ reproductive problems due to the adverse economic effect of reproduction failures (Kerslake et al. 2018). Therefore, it is likely that the dairy farm-workers are exposed during these activities.

Furthermore, the dairy farm-workers’ correct perception about the influence of poor milk yield and mastitis on culling of dairy cows resembles previous reports which noted milk yield losses as a risk factor for culling dairy cows (Orpin and Esslemont 2016; Horváth et al. 2017; Kerslake et al. 2018). Mastitis directly influences milk production in dairy farms and also increases production costs (Harmon 1994). Farm-workers’ well-informed perception could be to the diligence put on mastitis by dairy farms.

The dairy farm-workers’ perception that lameness is not a main reason for culling of dairy cows may be due to the fact that these conditions can be easily treated by the farm-workers (Borderas et al. 2008). Also, lameness is easily overlooked in pasture-based dairy farms; as such, an average of 25% of lameness cases are treated instead of culling (Borderas et al. 2008). Previous studies reported that lameness poses minimal culling risk (Beaudeau et al. 2000; Mason 2017). However, other studies mentioned that lameness induces poor milk yield, and poor milk yield is one major reason for culling (Fourichon et al. 1999; Jacobs and Siegford 2012). Lameness limits dairy cows’ locomotion and grazing ability hence the drop in milk yield. Therefore, dairy farmers may consider poor milk yield instead of lameness when culling dairy cows. As such, the farm-workers’ failure to identify lameness as a culling factor can be attributed to the minimal attention it receives.

Dairy cows are voluntarily culled at an average age of 6.5 years (Ansari-Lari et al. 2012; Adamczyk et al. 2016). Culling due to age is mostly affected by herd size, disease susceptibility, low fertility, and availability of replacement heifers (DeGaris and Lean 2008; Adamczyk et al. 2016). Voluntary culling is a dairy farm manager’s discretion that hardly involves the general farm worker’s input. Therefore, it is possible and logical for dairy farm-workers to overlook age when identifying factors responsible for culling and mortality.‬‬‬‬‬‬‬‬‬‬‬‬‬‬‬‬‬‬‬‬‬‬‬‬‬‬‬‬‬‬‬‬‬‬‬‬‬‬‬‬‬‬‬‬‬‬‬‬‬‬‬‬‬‬‬‬‬‬‬‬‬‬‬‬‬‬‬‬‬‬‬‬‬‬‬‬‬‬‬‬‬‬‬‬‬‬‬‬‬‬‬‬‬‬‬‬‬‬‬‬‬‬‬‬‬‬‬‬‬‬‬‬‬‬‬‬‬‬‬‬‬‬‬‬‬‬‬‬‬‬‬‬‬‬‬‬‬‬‬‬‬‬‬

Milk fever is a metabolic disease caused by excess blood calcium loss into milk and failure to absorb calcium from bone reserves, rumen, and intestines (DeGaris and Lean 2008). This disorder is common amongst high-producing dairy cows such as Holstein-Friesland. However, minimal clinical milk fever cases (1–10%) per calving season have been reported in dairy farms (Heringstad et al. 2005; DeGaris and Lean 2008). As such, milk fever is a common reason for culling and mortality but with a below par proportion (7.8%) (DeGaris and Lean 2008). Also, milk fever is treated with a calcium solution intravenous injection, thus lowering culling and mortality risk (McGuffey and Shirley 2011). So the dairy farm-workers’ failure to identify milk fever as a reason for culling and mortality can be accounted for by the perception that it is easy to treat milk fever and can be prevented by a proper dietary formulation.

Concluding, the study’s objective was to assess dairy farm-workers’ knowledge about culling and mortality. In this study, educational level did not significantly affect agriculture production knowledge and its application to advancing productivity. The study participants showed well-informed perceptions about culling due to reproductive failures and poor milk yield. However, they failed to identify lameness, age, and milk fever as reasons for culling dairy cows. The lack of training on dairy health, food safety, and limited exposure to farm information need to be addressed to improve farm-workers’ knowledge levels and enhance productivity. The study also provides baseline information for formulating mitigation strategies against unnecessary culling and mortality.

## Data Availability

The datasets generated during and/or analysed during the current study are available from the corresponding author on reasonable request.
